# A Comprehensive Review on Nutraceuticals: Therapy Support and Formulation Challenges

**DOI:** 10.3390/nu14214637

**Published:** 2022-11-03

**Authors:** Vivek Puri, Manju Nagpal, Inderbir Singh, Manjinder Singh, Gitika Arora Dhingra, Kampanart Huanbutta, Divya Dheer, Ameya Sharma, Tanikan Sangnim

**Affiliations:** 1 School of Pharmacy, Chitkara University, Baddi 174103, Himachal Pradesh, India; 2 College of Pharmacy, Chitkara University, Rajpura 140401, Punjab, India; 3 NCRD’s Sterling Institute of Pharmacy, Nerul, Navi Mumbai 400706, Maharashtra, India; 4 School of Pharmacy, Eastern Asia University, Pathum Thani 12110, Tanyaburi, Thailand; 5 Faculty of Pharmaceutical Sciences, Burapha University, Chonburi 20131, Muang, Thailand

**Keywords:** cardiovascular diseases, interactions, nutrition, prevention, therapeutics

## Abstract

Nutraceuticals are the nourishing components (hybrid of nutrition and pharmaceuticals) that are biologically active and possess capability for maintaining optimal health and benefits. These products play a significant role in human health care and its endurance, most importantly for the future therapeutic development. Nutraceuticals have received recognition due to their nutritional benefits along with therapeutic effects and safety profile. Nutraceuticals are globally growing in the field of services such as health care promotion, disease reduction, etc. Various drug nutraceutical interactions have also been elaborated with various examples in this review. Several patents on nutraceuticals in agricultural applications and in various diseases have been stated in the last section of review, which confirms the exponential growth of nutraceuticals’ market value. Nutraceuticals have been used not only for nutrition but also as a support therapy for the prevention and treatment of various diseases, such as to reduce side effects of cancer chemotherapy and radiotherapy. Diverse novel nanoformulation approaches tend to overcome challenges involved in formulation development of nutraceuticals. Prior information on various interactions with drugs may help in preventing any deleterious effects of nutraceuticals products. Nanotechnology also leads to the generation of micronized dietary products and other nutraceutical supplements with improved health benefits. In this review article, the latest key findings (clinical studies) on nutraceuticals that show the therapeutic action of nutraceutical’s bioactive molecules on various diseases have also been discussed.

## 1. Introduction

Nutraceuticals are characterized as ‘specially designed preparations’, formulated with the aim of fulfilling specific dietary requirements and/or offer preventive health care. Nutraceuticals are the formulation of nutrient/nutrients which helps in prevention and treatment of some diseases, in addition to a supplement diet. Nutraceutical is a term given by Dr. Stephen De Felice in 1989 and came from two words “nutrition” and “pharmaceutical”. These are foods or a part of foods that are beneficial in providing various health benefits including the treatment and/or prevention of the disease. Science of nutrition has increasingly achieved new horizons, starting from the anticipation of deficiencies in nutrients to prominence on human health and prevention and treatment of chronic ailments. Terms ‘nutraceuticals’, ‘food supplements’, ‘dietary supplements’ have evolved after the concept was originated by Dr. De Felice. There is no sharp demarcation between food supplements and nutraceuticals given by regulatory authorities. Literature of recent years emphasizes on redefining the concept of nutraceuticals, taking into consideration the efficacy, safety and toxicity of these products. Food products are nourishing substances that are eaten, drunk or otherwise taken to sustain life, provide energy and promote growth. Currently, isolation of nutrients from these food products are well recognized and used. The starting point to differentiate food/dietary supplements and nutraceuticals is the identification of an epidemiological target, followed by safety and efficacy studies that understand the mechanism of action. One approach to differentiate these two types of formulations is describing ‘food supplements’ as agents to compensate deficiencies in micro- or macronutrients; in addition, the use of a “nutraceutical” in the treatment of a pathological disease must be supported by strong scientific evidence [1]. With adequate clinical evidence, nutritional supplements should have a strong safety profile with few undesirable side effects and better bioavailability. There is a very fine line of demarcation between two type of formulations: the same ingredients may work as a nutraceutical or food supplement, but may be demarcated on the basis of claims. Nutraceuticals include single or combinations of pro- and pre-biotic foodstuff and food for special medical uses; and food supplements includes single or combinations of mineral, vitamins, protein supplements, functional foods and herbal products. By prolonging or eliminating the need for pharmaceuticals in subjects to fit for an alternative nonpharmacological treatment to a pathological condition, the incorporation of nutraceuticals into daily diet may aid in the prevention of pathological disorders. There are claims that foods including spices and herbs possess the tendency to decrease the risk of many diseases and can be highly beneficial in improving the quality of life [2]. There is a plethora of benefits that nutraceuticals have provided, including their promising results in the prevention and treatment of complicated diseases. However, there is a need of administration and prescription of nutraceuticals and they should be strictly regulated in order to prevent their uncontrollable use and side effects [3]. Several researchers have studied drug compound-based nutraceuticals to improve the efficacy as well as bioavailability. The safety and efficacy of various statins have been used in the prevention of cardiovascular diseases even in pregnant women. Nutraceuticals with an effective safety profile and well-established impact onpregnancy might be a suitable therapeutic option for preventing diabetes mellitus and hypertensive disorders, or as an adjuvant to therapy with standard medications. Calcium, omega-3 polyunsaturated fatty acids, vitamin D, folic acid, resveratrol, alpha-lipoic acid, zinc, inositol, and probiotic supplements are potentially proven candidates as novel nutraceuticals [4]. Researchers have evaluated the nutraceutical associated with the drug compound ezetimibe for patients atrisk of elevations of statin level, which further leads to cardiovascular diseases [5]. The use of a novel nutraceutical in blend with non-steroidal anti-inflammatory drugs (NSAIDs) has been proven a potential candidate for osteoarthritis, thus improving its efficacy and safety for commercial use [6].

The market remains robust and ever-growing for nutraceuticals such as antioxidants; omega-3 fatty acids; plants such asalgae, aloe vera, seaweed, and wheatgrass; teas and herbs such asginseng and Echinacea. A detailed findings including clinical data on nutraceuticals are shown in Table 1 [7,8]. A recent survey suggested that the nutraceutical market is expanding globally and the probability states that it may reach up to $340 billion by the year 2024. The compound annual growth rate (CAGR) of nutraceuticals is estimated to be 7.2% in the year 2016 to 2024. This increment in the growth of the nutraceuticals-based industry is associated with various factors such as a rise in demand for nutraceuticals, an awareness among people for the benefits of nutrition and an incremental rate observed in the healthcare graph [9,10]. Currently, Europe, USA and Japan account for ˃90% of the total global nutraceutical market and the global market is supposed to reach $336 billion by 2023 from $247 billion in 2019 at a CAGR of 8%. With this attainment of maturity of global markets, now the focus of nutraceutical players has been shifted towards developing economies, especially those across Asia Pacific, including India. The Indian market had only 2% market share of total global nutraceutical market in 2017. It is estimated to reach $11 billion by 2023, increasing at a CAGR of 21%. By 2023, India is also expected to hold at least 3.5% market share of the global market [11,12].

The COVID-19 virus is having a knock-on effect for every industry. The long-term repercussions are hard to predict as of yet. In a post-COVID-19 pandemic situation, the global economy is still struggling and its impact on the global trade and nutraceuticals market is rising with rapid deterioration in the supply of products irrespective of high demands. Nutraceutical industry is not able to bridge the gap between high demand and low supply as 75–80% of raw material used in nutraceuticals was sourced from China in North America, Europe and Asia Pacific. Due to China’s manufacturing shutdown, and the sudden recessive conditions in native regions, the nutraceutical production has reduced. The sharp surge in demand was observed for dietary supplements from consumers, as they provide a strong immune function and decrease possible health threats [25].

However, an officially shared and accepted definition of nutraceuticals is missing; these are also referred as ‘pharma food’—a powerful toolbox that is beyond the diet but before the drugs. Various formulations containing macronutrients (required in large amounts, e.g., omega-3 fatty acids, magnesium, potassium and calcium) [26], micronutrients (required in lesser amounts, e.g., minerals or vitamins) [27] and phytochemicals that are present in the food source [28] and are available at drug stores. Further, probiotics [29], minerals [30], polyunsaturated fatty acids [31], carotenoids [32], amino acids and proteins [33], vitamins [34], dietary fibers [35], spices and phytochemicals [36] have also become part of these formulations. Nutraceuticals exist as various types and may range from herbal products to isolated nutrient diets and may go up to existing genetically modified foods [37,38,39]. Plant foods such as vegetables, whole grains and vitamins are rich in dietary phytochemicals. Dietary supplements are consumed as such, or as isolated active ingredients. These phytochemicals are immensely diverse and these include carotenoids [40], phenolics [41], alkaloids [42], organosulfur [43] and nitrogen containing compounds [44]. However, these products may not be substantiated by scientific data on their safety, efficacy and effect on health and/or pathological conditions.

**Figure 1:**
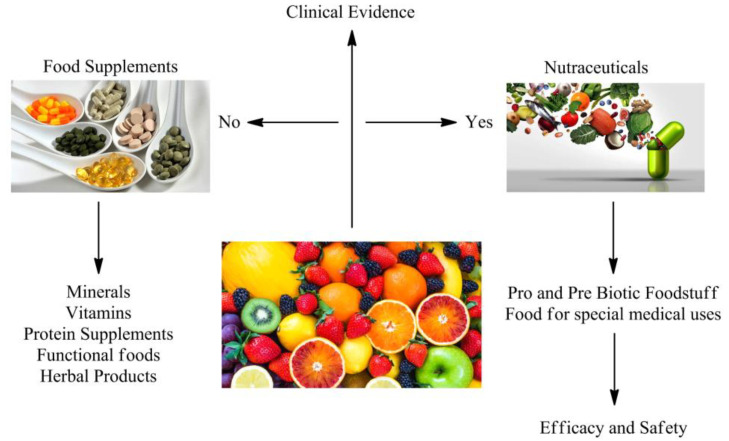
Potential roles of nutraceuticals.

In the pharmaceutical industry, it is mandatory to do clinical tests on animals or in vitro for the verification of a compound’s effects. On the contrary, in nutrition, there was no such method in the past for the verification of effects of foods in preventing or treating diseases. In recent years, however, the food composition has been scientifically tested and verified as people are becoming more and more aware of health-related issues and how food can directly or indirectly be responsible for maintaining proper health and preventing diseases (Figure 1) [45,46].

Nutraceuticals provide their benefits in a wide range of therapeutic areas such as cough and cold [47], anti-arthritis [48], digestion [49], sleeping disorders [50] and treatment of cancers [51], depression [52], diabetes [53], cholesterol [54], blood pressure [55] and pain killers [56]. The research and development sectors for nutraceuticals are working at their peaks to discover how various nutraceuticals can prove to be of significance in the pharmaceutical industry. Scientific needs for nutraceuticals demand standardization of the constituents and cautious development of protocols and implement clinical studies which will form the foundation for consumer health and impact on nutraceutical companies [57,58].

In the last 10 years, a huge growth has been observed in the awareness of nutraceuticals and their use as powerful therapeutic supplements. Nutraceutical medicine has now been accepted as a part of Complementary and Alternative Medicine (CAM) and, thus, it has been incorporated as a new branch of CAM [59,60].

Due to dynamic action of nutraceuticals (nutritional and medicinal action), their popularity amongst general public and healthcare providers has increased over medicines. A current review comprehensively discusses the use of nutraceuticals in preventive and support therapy, followed by compiled literature on patents published on said topic.

## 2. Nutraceuticals in Various Diseases

**Figure 2:**
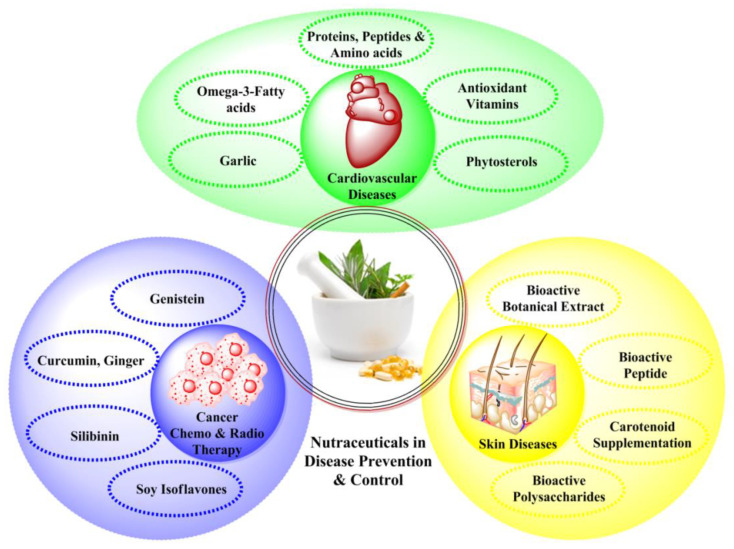
Nutraceuticals and Dietary Supplements in various diseases.

Nutraceuticals help enhance health, wellbeing and modulating immunity, thus preventing and treating various diseases and health issues (Figure 2) [61]. There are a variety of diseases that can be treated with the help of nutraceuticals which are discussed below:

### 2.1. Nutraceuticals in Cardiovascular Diseases

Among all other diseases, cardiovascular diseases reveal significant risk-related factors acquiescent to nutraceutical intervention [62]. There is significant evidence indicating that nutraceuticals can be used in cardiovascular diseases [63,64].

Cardiovascular diseases (CVDs) mainly affect the blood vessels and the functioning of the heart. CVDs are one of biggest causes of mortality, as they account for about 30% of deaths all over the world annually [65]. Dietary supplements have been proven to be beneficial in risk management and prevention of cardiovascular diseases and can be classified broadly into the ones used in the treatment of arrhythmias [66], Congestive heart failure [67], angina [68], hypertension [69] and hyperlipidemias [70,71]. Some of the nutraceuticals and dietary supplements that are used for the treatment and prevention of CVDs are discussed below.

#### 2.1.1. Allicin and Alliin

Ischemic heart disease and atherosclerosis are associated with elevated levels of plasma triglycerides and blood-cholesterol are associated with. *Allium sativum* is antihyperlipidemic in nature and it exerts its effects by the elimination of cholesterol and its end-products in high amounts in the feces and by decreasing the cholesterol synthesis endogenously [72]. This helps in producing more favorable ratio of HDL and LDL. Allicin and alliin can effect cholesterol levels, if these can be protected by gastric acids. Garlic supplementation on serum cholesterol was assessed using thirteen placebo-controlled trials concerning 781 patients. Garlic also has some inherent antihypertensive effect, apart from being antihyperlipidemic [73,74].

#### 2.1.2. Omega-3Fatty Acids

Omega-3 fatty acidsare derived from marine sources and are called polyunsaturated fatty acids (PUFAs). Docosahexaenoic acid (DHA) and Marine omega-3 eicosapentaenoic acid (EPA) plays a critical role in the treatment and prevention of cardiovascular diseases. In one study, it has been reported that intake of fish oil supplements reduced mortality rate by 29% for over a period of 2 years in the diet and reinfarction trial (DART), which was a randomized trial involving 2033 men post-myocardial infarction. Consumption of fish oil led to a noteworthy reduction in unexpected demises by 45%, cardiovascular diseases deceased by 30% and a 20% decrease in overall mortality. According to the recent clinical trial studies, it has been found that the risk of cardiac arrhythmias is decreased with the help of omega-3 fatty acids, and they also improve the health of the patients suffering from plaque formation caused by atherosclerosis. Omega-3 fatty acids enhance the electrical-stability of heart cells, thereby extending its relative refractory period and helping treat arrhythmias [75,76].

#### 2.1.3. Soy Isoflavones

Soy proteins and soy isoflavones are important nutrients with potentially medicinal benefits such as antihyperlipidemic, antihypertensive, anti-hyperglycemic, antioxidant, anticancer, anti-inflammatory, anti-obesity and neuroprotective activities that support the biological plausibility for observational associations. It is evident from clinical study reports that consumption of soy protein reduces serum cholesterol levels in humans [77]. In addition, USFDA has evidenced that 25g of soy proteins or isoflavone per day intake showed a lowering of blood pressure in postmenopausal women. Moreover, soy proteins exert favorable effects on the serum lipid concentrations, especially in hypercholesterolemic patients. In a study, subjects fed with a diet low in saturated fat leads to the decrease in risk of coronary heart disease. Soy isoflavone had no effects on the lipid profiles [78]. Further a study reported significant reduction of the ratio of LDL to HDL after intake of new soy products, having high levels of isoflavones, cotyledon soy fiber and soy phospholipids (Abaco and Abalon) [79].

#### 2.1.4. Proteins, Peptides and Amino Acids

Hypertension is associated with cardiovascular diseases. ACE (angiotensin converting enzyme) inhibitors have been a chief line of therapy to treat the condition, but these drugs lead to side effects such as hypotension, elevated levels of potassium, impaired renal function, coughing and skin rashes [80]. Natural, ACE inhibitors are found in casein and whey protein derived from milk. It is also evidenced from animal studies that these milk-derived proteins exert antihypertensive effects. The same has been reported in clinical studies where a statistically significant hypotensive effect has been observed [81].

#### 2.1.5. Antioxidant Vitamins

Antioxidants have been used as potential supplements in chronic diseases such as cardiovascular diseases and cancer. They reduce LDL-cholesterol oxidation by counteracting the damaging effects of free radicals. Vegetables, fruits, fish and fixed oils contain antioxidant vitamins in large volumes, which works by preventing the formation of oxygen free radicals or by entrapping them. It has been evidenced in some epidemiologic studies of CHD patients on a diet of large quantities of antioxidants that they result in fewer incidences of morbidity and mortality. Supplements having antioxidant vitamins C and E help in preventing CHD. However, supplementation with ß-carotene can produce adverse effects and, thus, is not recommended. The National Health and Nutrition Examination Survey-I cohort study observed that risk of CHDs decreases in vitamin C intake, which was found in the observation of over 10 years in American men and women of age group of 25–74 years, in which subjects were randomized with diverse combinations of 10 nutritional supplements for over five years [82].

#### 2.1.6. Phytosterols

Phytosterols are structurally similar to cholesterol. They tend to compete for absorption through the small intestine. These are found naturally in vegetable oils, seeds, nuts, grains, wood pulp, etc. [83]. Intake of phytosterols led to increased hepatic uptake of LDL, reduced blood LDL levels, and reduced absorption of cholesterol. Studies have indicated up to 15% reduction in LDL levels by intake of plant sterols [84]. Plant sterols are derived from natural grains such as soy, sunflower and corns. Various studies evidenced that the consumption of 2–3 g/day of plant sterols/stanols tend to reduce LDL cholesterol levels up to 20%; although, there is substantial variation amongst individuals [85].

### 2.2. Nutraceuticals in Cancer Chemo- and Radiotherapy

Radiotherapy and chemotherapy are conventional therapies for cancer therapy but have serious side effects and various complications (e.g., pain, fatigue, diarrhea, vomiting, nausea and hair loss) [86,87]. There are some cancers that are highly resistant to chemo- and radiotherapy and, because of this, systemic cytotoxic chemotherapy and radiotherapy are not very operative at cultivating patient subsistence [87,88]. In this situation, various combination therapies overlay an efficient means to treat cancer. Likewise, there are a variety of plants and natural supplements that are observed to reduce side effects of radiotherapy and chemotherapy. Thus, these should be used in the combination with radio- or chemotherapy for the reduction of side-effects and to augment treatment effectiveness. Proliferation of cells leading to cancer results in the need to treat, and the nutraceutical industry is evolving to treat the needs of the consumers. The evolution of the nutraceutical industry can be recognized as it has now reached disease prevention after it started from health promotion. A plethora of currently used herbs and phytochemicals are safe pharmacologically and have been proven to be potent nutraceuticals in suppressing tumor progression, alleviating the disadvantages of radio- and chemotherapy and increasing the sensitivity of these therapies [89,90]. Caponio and his team evaluated the effects of the phenolic compounds found in Aglianico Grape pomace (GP) on colorectal cancer cell lines at varying stages of development after subjecting them to an in vitro digesting model. Aglianico GP extract was found to have strong effects on cell proliferation and apoptosis, as well as on other cellular processes. A substantial upregulation of Bax, as well as the Bax/Bcl-2 ratio and caspase-3, was observed in both HT29 and SW480 cells. UHPLC-DAD analysis revealed that anthocyanins, phenolic acids, and flavonoids were the primary components responsible for the elevated (total phenolic content) TPC and antioxidant activity in the Aglianico GP digested extract [91]. In 2021, Zhang and his co-workers studied the combined effect of chrysin and apigenin by suppressing the activity of P38-MAPK/AKT pathway in colorectal cancer. Apigenin and chrysin, both at 25 µM, substantially inhibited clone number, migration and invasion, while increasing apoptosis in both colorectal cancer (CRC) cell lines. Additionally, chrysin and apigenin significantly suppressed p-P38 and p-AKT. Anisomycin, a P38 agonist, effectively mitigated the tumor-inhibiting action of apigenin and chrysin. Together, apigenin (25 µM) and chrysin (25 µM) had a synergistic impact in limiting the proliferation and metastasis of CRC cells by suppressing the P38-MAPK/AKT pathway [92].

#### 2.2.1. Curcumin (Diferuloyl-Methane) from Turmeric (Curcuma Longa)

Curcumin has been classified as a commanding nutraceutical for cancer treatment. Pre-clinical studies with curcumin suggest that it inhibits carcinogenesis in different types of cancers, such as pancreatic, colorectal, prostate, gastric and hepatic cancer; in addition, it has been able to suppress it at every step, that is, angiogenesis, metastasis and proliferation. It is much more effective when it is in combination with the chemo- and radiotherapies for cancer treatment [93,94].

#### 2.2.2. Ginger

Ginger is an antimutagenic, antioxidant and anti-inflammatory nutraceutical and is known to diminish the side effects of radio and chemotherapy. It is these properties of ginger that provide helpful radio-protector activity. A reported loss in the doses of morphine in cancer patients is seen with the help of Ginsenoside Rf and Ginseng and its polysaccharides are helpful in reducing the side effects of cancer treatment therapies, which has reported to cause a 50% less risk in the recurrence of cancer [95].

#### 2.2.3. Genistein

Genisteinis a potent isoflavone and has promising anti-carcinogenic properties. In vitro studies have shown that there are a few components that exert their antitumor effects only at higher concentrations that are not possible to achieve at normal dietary consumption [96]. Thus, it is difficult to achieve the desired effect at the tumor site, which leads us into thinking that the mode of delivery is a very important factor that needs to be considered in in-vivo studies and clinical trials. For a therapy to be formulated, the non-toxicity of the natural components is a very important factor. Nevertheless, it has been found that some compounds are more potent if administered early in life and Genistein is one of them [97].

### 2.3. Nutraceuticals in the Treatment of Prostate Cancer (PCa)

Prostate canceris the most common type of cancer and has been recorded as the second leading cause of mortality by cancer in American males. It has been noticed that men descendent from United States and Africa have the highest prostate cancer mortality rates compared to those of European descent. Although current strategies of treatment are quite potent and effective, there are always opportunities of resistance towards disease and progression to metastasis and many more that may develop over time. Therefore, more effective and non-toxic therapeutic approaches are required to overcome these major hurdles and provide proper management and treatment of this disease. Thus, in this regard, various potential safe nutraceuticals are available as effective anti-PCa agents. Evidently, execution of nutraceuticals might help in the development of precision in the design of the medicines and reduce the toxicities associated with chemotherapy and decrease the resistance of disease and have the potential of treating the disease in both localized and advanced stages. Some of the nutraceuticals that can potentially be used as treatment are discussed below [98,99].

#### 2.3.1. Silibinin

Silibinin that is, flavanolignan from milk thistle “Silybummarianum” seeds have potent anti-carcinogenic effects for a plethora of tumors including PCa. A pre-clinical animal model shows significant anticancer activity of silibinin in the treatment of PCa, and phase II clinical trials bioavailability studies have also been evaluated. There is still a need of larger clinical trials to be performed to confirm the biological efficacy and effectiveness of silibinin as a nutraceutical for effective clinical management of advanced or localized form of PCa [100,101].

#### 2.3.2. Soy Isoflavones

Soy Isoflavones are members of the polyphenolic flavonoid family, mainly found in soybeans, red clover, kudzu root, etc., and are widely used in cuisines from Asia and Africa. Clinical studies have proven some benefits against the disease PCa with the help of Soya Isoflavones as it has a marked effect on inflammatory signaling and insulin. Among others there have also been reports in which Isoflavones have shown immuno-modulatory properties in the plasma of asymptomatic bio-chemically-recurrent PCa patients. It was found out that the high concentration of genistein in plasma was linked to a 69% reduction in the risk of future development of PCa in Chinese patients. If isoflavones are administered short term, such as for a period of 6 weeks, they show an inhibitory effect in the cell cycle of prostate tumor and also shows apoptotic-associated signaling; however, it does not have any influence on the levels of testosterone, PSA, free testosterone and total cholesterol in patients suffering from PCa. A clinical trial performed for 6 months showed that the intervention of soy protein had no effect on molecular markers determining proliferation and apoptosis, i.e., EGFR, Bax:Bcl-2, Bax:PCNA ratios in patients with high-risk and low-grade PCa. On the contrary, soy protein (alcohol-washed) intake decreased the tissue levels of Bax and PCNA in comparison with patients receiving milk protein treatment [102,103]. Recently, Zhu and his team reported that the potential cholesterol-reducing effects of soy protein isolate were enhanced by glycation with soy soluble polysaccharide at higher degrees of glycation (DG) [104].

### 2.4. Nutraceuticals for Skin Treatment

The skin is known to be the body’s largest organ and it offers protection against all sorts of microorganisms, ultraviolet radiations and chemicals also participating in sensitivity. As a result of having a major role in protecting the body, skin may face alterations, such as immune dysfunction, photo-aging and inflammation, which may result in harm on human health [105]. A potential strategy of delaying or diminish pre-mature ageing of the skin and alleviation of skin-related disorders can be found with the help of nutraceuticals. These nutraceuticals can be bioactive peptides, bioactive polysaccharides, botanical extracts, carotenoids, etc. Supplementation with these products in several human trials has evidenced fewer signs of ageing and also protection against UV-radiation ageing [106].

#### 2.4.1. Bio-Active Peptides

Peptide moieties are the combination of two or more amino acids and are short sized with low molecular weight (<3 kDa), and some may perform important biological actions thatare termed bioactive peptides. Bioactive peptides have been isolated from a wide variety of dietary proteins, including plant and animal. Eggs, milk (casein and whey), and meat proteins are the most common sources of animal protein. Soy, oat, pulses (chickpea, bean, pea and lentil), canola, wheat, flaxseed and hemp seed are common plant sources for bioactive peptides [107]. Peptides that are used for cosmetic purposes are usually derived from collagen and typically serve as nutraceutical formulations because of their increased bioavailability and solubility [108]. It has typically been observed in a controlled study of VERISOL^®^, which contains bioactive collagen peptide (BCP). In this study VERISOL^®^ and a placebo were given to subjects for 8 weeks and skin wrinkles were measured before the treatment and after 8 weeks. It was observed that BCP promoted a significant decrease in the eye-wrinkle volume in comparison to placebo after a period of 8 weeks of treatment. Not only this, BCP intake showed an increase in the content of elastin and procollagen type1 along with an increase in the fibrillin content. Thus, this treatment reduced wrinkles and has encouraging effects on skin matrix synthesis [109].

Peptan F and porcine origin Peptan P are some other nutraceuticals of fish origin containing collagen peptides used to slow aging by effectively maintaining the moisture content within skin layers. Recently, a study indicated improved skin properties without risk of oxidative damage by use of a nutraceutical product Celergen^®^, proving it a safe and effective supplement. This nutraceutical is based on a marine collagen peptide derived from deep sea fish, grape skin, coenzyme Q10 and leutonin [110,111].

#### 2.4.2. Bio-Active Polysaccharides

These are sugar-based polymers that have the energy storage and structural functions. They are present in life forms such as plants, fungi, animals and prokaryote organisms having diverse monosaccharide combinations, physicochemical properties and structures. The most useful of them for the nutraceutical formulations is the Glycosaminoglycans from the marine origin. The basic unit of these are- an un-branched disaccharide (repeating) unit of amino sugar called *N*-acetylglucosamine or *N*-acetylgalactosamine and an uronic acid called glucuronic or iduronic acid [112,113]. A human trial of the formulations containing these was conducted using Imedeen^®^ DermOne^®^, these contain some protein fractions as well along with the glycosaminoglycans and served as dietary supplements for skin care. In addition to the protein the supplement contained zinc gluconate and vitamin C which are relevant for skin care. In the trial conducted, 10 women were treated with an amount of 500 mg of Imedeen^®^ for a period of 90 days. Parameters evaluated were, dryness, brittleness of hair and nails, wrinkles and mottles. It was seen that after 90 days, all these signs were improved, and observations established skin thickness and elasticity [105].

#### 2.4.3. Bio-Active Botanical Extracts

These extracts are multifaceted mixtures of various compounds having diverse structures and origin. Since long times they are being used and reviewed. Polyphenols are one of the key natural compounds with cosmetic applications with a plethora of families and structures. These are plant-based micronutrients available from diet. These are beneficial as support therapy in the prevention of diseases and also improved the outcome of diseases. Various polyphenols have significantly different bioavailability and the most abundant polyphenols in our diet possess the maximum concentration of active metabolites in target tissues. Their composition and proportion vary depending on the procedure of extraction and families [114]. Pycnogenol^®^ is a formulation being made with the help of these and is rich in catechins, flavonoids and procyanidins (B1, B2, B3, B7 C1 and C2), also, they contain phenolic acids such asferulic acid and caffeic acids. It is also confirmed to have several effects such as cholesterol lowering and cardiovascular benefits because of its antidiabetic, anti-inflammatory and antioxidant properties [115,116,117].

#### 2.4.4. Carotenoids

These are naturally occurring pigments found in algae, photo-synthetic bacteria and various plants. These have linear tetra terpenoid structure. These are found in natural sources such as fruits, vegetables etc. α-carotene, β-carotene, β-cryptoxanthin, lutein, zeaxanthin, and lycopene are the most commonly used dietary carotenoids [118]. These carotenoids are used for skin health such as anti-ageing and photo protection of skin. The probiotics and carotenoids are reported for decreasing the skin damage due to UV-exposure and also in modulating early skin biomarkers of UV effects. A Carotenoid mixture supplement of α-carotene, β-carotene and lutein are proved effective in photo protection. Similarly, a mixture of beta-carotene, lutein and lycopene carotenoids is reported for protection against erythema. The vitamin C and E is studied for the photoprotective effect and found to be effective in skin health care [119]. Vitamin C is a hydrophilic vitamin, commonly taken in large doses via consuming various food products with the intent of inhibiting the formation of carcinogenic nitrous metabolites. It acts as a cofactor for the synthesis of collagen fibers and inhibits the biosynthesis of elastin in fibroblasts thereby preventing its accumulation, which is highly present in photo-damaged skin. In combination with vitamin E, it acts synergistically working with its mechanism of transformation. Vitamin E is the main lipophilic antioxidant and is found in the form of tocopherols. It binds with peroxyl radicals, thereby preventing lipid peroxidation of polyunsaturated fatty acids. In addition, itsuse for preventing photodamage, sunburn, atopic dermatitis, etc., is clearly evidenced [120,121].

### 2.5. Nutraceuticals as Specialized Medical Products

According to the legal basis, dietary foods and enhancements for distinct medical purposes are specialized medical products. These dietary supplements should be regulated according to the regulatory agencies such as ‘European Food Safety Authority’ and the ‘U.S. Food and Drug Administration’, in addition to numerous national protocols issued most often by the ‘Ministry of Agriculture’ and/or ‘Ministry of Health’ of various countries around the world [122].

**Figure 3:**
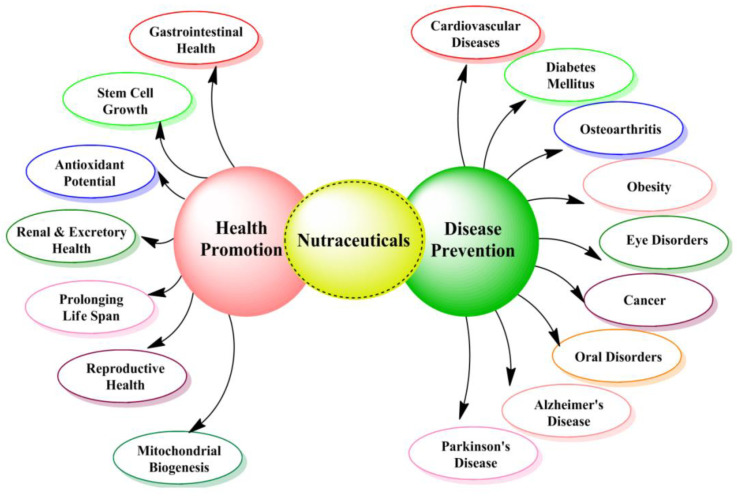
Role of nutraceuticals in disease prevention and health promotion.

Nutraceuticals are non-specific biological therapies used to promote wellness, prevent malignant processes and control symptoms. Figure 3 shows a flow chart indicating role of nutraceuticals in health promotion and disease prevention. Various nutraceuticals in health promotion are summarized in Table 2 [123,124,125].

## 3. Formulations and Challenges Involved

A quality nutraceutical formulation with physical and chemical stability, adequate safety, technological feasibility and still cost effective entails many challenges. When compared with drug molecules that are well defined chemical entities, botanicals are complex ingredients containing multiple chemical constituents and usually several classes of compounds are present in a single product. Most of these botanicals are susceptible to heat, light, oxygen, alkaline pH and elevated humidity. These are usually having poor flow, bulk density and variable particle size distribution. Thus, successful development of nutraceutical formulation requires knowledge of the fundamental aspects of the physicochemical properties of the different types of ingredients, the use of adequate techniques of manufacturing, selection of the right excipients and the addition of suitable manufacturing overages based on critical stability studies [126,127]. Here, emphasis is given on:

- Challenges with various dosage forms;
- Approaches to deal with formulation challenges;
- Excipients selection.

### 3.1. Challenges in the Formulation of Nutraceuticals and Dietary Supplement

While formulating nutraceuticals, the poor aqueous solubility, high melting point of nutraceuticals and chemical instability of active constituents pose difficulties. For example, omega-3 fatty acids, carotenoids, oil soluble vitamins, curcumin possess high nutritional value but are poorly soluble. Therefore, the possible approach is to formulate these as novel delivery systems. These novel delivery systems make them costly. Thus, efforts are needed to make these formulations cost effective [128]. 

Another challenge in formulating nutraceuticals is their high melting point. For example, phytosterols, fatty alcohols and carotenoids all have high melting points that may cause instability to formulation. Therefore, the possible approach is to prepare solid dispersion/dissolve in suitable grade solvent and introduce in food as suspended nanocrystals. However, the challenge is again that it leads to deteriorated stability and shelf life, disagreeable appearance, and obnoxious odor and mouthfeel, which affect market value and customer demand. Therefore, there is need to develop cost-effective technologies [129,130,131]. 

Chemical instability is another challenge. For example, omega-3 fatty acid rich oils, such as fish oils, flaxseed oil, cod liver oils; carotenoids; lycopene or curcumin all have stability issues. The extent of the chemical degradation is completely dependent on the composition of the bioactive product, the environmental conditions such as temperature, pH, pressure, etc., or the presence of metals or other such oxidation-promoting agents. For such compounds, the development of nanoscale products is essential to protect them from degradation [132,133,134,135,136,137,138]. Further, in the case of the development of probiotics, special, selective bacterial strains are necessary. A current challenge is the selection of the proper strain followed by their incorporation in foods. Application of any mismatching bacterial or toxic cultures, in addition to their negligent handling, can cause disastrous consequences. Apart from these challenges, other considerations are solid dosage formulation and process design for drug products and nutrition products that are similar, but the purpose and regulatory requirements may differ [139,140].

Finally, there is a challenge in the formulation of nutraceuticals and dietary supplement dosage forms that are suitable for different groups of the aging population, especially older adults and children. This is because this group of people has limitations in solid dosage from (tablets or capsules) swallowing (dysphagia). Therefore, advanced dosage forms such as orodispersible tablets, fast dissolving films and easy-swallowing gels, which are normally used in pharmaceutical applications, have to be considered in nutraceutical and dietary supplement administration [141,142,143].

### 3.2. Approaches to Deal with Formulation Challenges

One of the most widely used approaches is isolation or preparation of concentrates of nutraceuticals from natural sources. It is advantageous as most of herbal nutraceuticals are to be administered in a large dose per daily serving. Further, multiple “active ingredients” are present in different sources. There is significant variation in active ingredient compression and flow characteristics within one dosage form. There are large variations in heat and moisture sensitivity of ingredients within one formula. Significant stability challenges are there with multiple opportunities for interaction. Different extraction processes involve microwave-assisted extraction, counter current extraction, maceration, percolation and Soxhlet extraction. Natural bioactive compounds are plant extracts; herbal concentrates; fruit, vegetable and specialty concentrates; and fungal and microbial materials, which are included in feedstock to produce concentrates (bio-fermentation process). Concentrates may include excipients used in production (such as spray dried carriers) [144,145].

Another promising approach is modification in delivery systems. Novel Drug Delivery Systems have been used to modify the properties of various compounds to produce new generations of drug compounds. They also play a role in the food industry and in various kinds of nutritional supplements. Various formulations of supplements prepared by nanotechnology, called nano formulations, that have increased bioavailability, reduced side effects and protect the active ingredients against the process of degradation, have been reported [146,147]. The polyphenols obtained as nutraceuticals in the form of dietary sources have proved greatly advantageous in improving diseased symptoms as observed in preclinical and clinical studies. One of the most commonly encountered disadvantages is its lower bioavailability due to the lesser gastrointestinal absorption in the upper tract attributable to hydrophobicity, presence in the polymeric or glycosylated form in foods, and they bound tightly with the food matrices. All these factors lead to the minimum bio-accessibility of polyphenols inside the body. Therefore, food processing-based research and development techniques along with nanoformulations, enzymatic treatment, probiotics combination therapies, among others, have proved advantageous to overcome this challenge [148,149]

#### 3.2.1. Liposomes and Nanoemulsions

Liposomes and Nanoemulsions are also termed as bilayer phospholipid vesicles and possess greater potential for the industry of nutraceuticals as they can simultaneously encapsulate both hydrophilic and lipophilic materials. This ensures a synergistic effect and is also helpful in the protection of bioactive compounds that are highly sensitive, ensuring enhanced bioavailability, sustainable release and storage stability. As the nanoliposomes have unique properties, they can be effectively used in operative prevention against diseases and also for health promotion. One of the latest examples is lipid-based nanocarriers, nanophytosomes, which help in enabling the delivery of botanical nutraceuticals. They have the potential to be used in various food products for the design of novel functional beverages and food products. A study indicates increased chemical and physical stability of rutin when rutin complexes were formed in the form of phytosomes. These phytosome complexes of rutin were formed with the help of phosphatidylcholine (PC) with the molar ratio of (rutin: PC) being (1:3) with a particle size of less than one hundred nm and 99% encapsulation efficiency, as it was able to mask the undesirable properties of rutin. These were termed as phytosomes, phosphatidylcholine (PC)—rutin complexes [150,151].

#### 3.2.2. Lipid-Based Carriers

Lipid formulations in the form of nanocapsules, micronized carries are potential candidates to be used to effectively enhance the controlled release, solubility and bioavailability of phenolic compounds. As an example, β-Car nanocapsules (>300 nm), due to their physical stability showed only negligible variations during storage, which suggested that they can be widely used as functional foods and beverages along with being nutraceutical products [150,152].

#### 3.2.3. Polysaccharide Matrices

These are the kind of matrices that have numerous enzymatic exposures that guarantee degradation at specific points in the large and small intestine. When being used as a nanoparticle coating, they can effectively retard the nonspecific release of bioactive components encapsulated within until the coating is exposed in the intended environment where it was intended to be released. These coated nanoparticles can potentially be used to target various diverse organs of the GI tract to help improve the oral bioavailability [150,153].

### 3.3. Excipient Selection

Next, the approach to deal with formulation challenges of nutraceuticals is modification in formulation parameters by appropriate selection of excipients. For nutrition, the final formula must be robust to accommodate the variable physical characteristics of natural ingredients in a complex formula. Materials and manufacturer must meet internal quality and safety, specifications and performance requirements. One formulation’s functionality can be another formulation’s dysfunctionality. Excipient functionality can only be properly assessed in the context of a particular formulation and manufacturing process. For natural product formulation, excipient functionality in a particular formula is heavily influenced by the complex combination of multiple active ingredient characteristics. Occasionally, seemingly equivalent excipients are not equivalent in functionality [154].

## 4. Safety and Quality Control of Nutraceuticals

Nutraceuticals are taken as supplements by users, which are available as over-the-counter products. Therefore, their safety is of prime concern, else it may lead to lethal effects. The most commonly observed issues are contamination, adulteration (inadvertent or intentional) or misleading labels. To demonstrate adulteration, three different detection strategies may be adopted as (1) the presence of an undeclared substance, (2) that a component is deviated from its normal level (content) and (3) that a profile is unlikely to occur [155].

Adulteration may be inadvertent adulteration or intentional adulteration. Inadvertent adulteration may exist due to different conditions. For example, during the different stages of plant growth, the formulation and manufacturing of nutraceuticals, or during storage, contamination with fertilizers, heavy metals, fertilizers or microbial agents may take place. Adulteration may also take place with synthetic drugs, substitute species, dust, pollens, insects, rodents, parasites, microbes, fungi, mold, toxins and heavy metals. Any of this type of contamination may lead to infections or even serious illnesses such as gastritis and associated complications, liver injury and even life-threatening conditions. Therefore, raw-material and finished-product quality control is required and may be determined by specifications outlined in certain monographs, along with stability of active compound(s) and microbiological control [156].

Serious harmful effects may result from the intentional adulteration in supplements or herbal remedies. It usually occurs with synthetic compounds, mostly undeclared. It usually occurs with the intentions of altering pharmacological response and earns economic benefits. The sourcing of nutraceuticals from plants is often very limited, and extract preparation is time- and cost-consuming [157,158]. Therefore, these adulterations are not allowed from regulatory authorities.

Some examples of adulterations are quoted in below sections.

(a) Ibutramine hydrochloride monohydrate is a drug molecule that works by inhibiting serotonergic and noradrenergic reuptake and shows effects as an anti-obesity drug, and is a common adulterant. In a study conducted with twenty-two samples of dietary supplements in China, eleven were found to be contaminated with phenolphthalein, *N*-mono-desmethylsibutramine, and sibutramine. In another similar study performed on fifteen samples in China, four of them contained sibutramine and *N*-di-desmethylsibutramine [159]. Further, it has also been reported that two pregnant women in Turkey lost their wombs due to consumption of adulterated Chinese herbal medicine “meizitanc” [160]. Sibutramine has also been reported as a solvent in slimming preparations. It has led to mania-like psychosis in two women in Hong Kong [161].
(b) Fenfluramine is another dug that was used as an adulterant in Chinese traditional medicines and found in many slimming preparations. It caused primary pulmonary hypertension and valvular heart disease. This drug was withdrawn from market in 1997 [162].
(c) In some weight control programs using an orexigens, diuretics, stimulants and laxative agents, it has been demonstrated that these products contain adulterants as ephedrine, norephedrine, caffeine and furosemide [163].
(d) Morphological substitute usage is another common example of adulteration that may cause serious health issues. For example, *Panax ginseng* (Araliaceae), also known as “Asian or Korean ginseng”, is used as traditional medicine. It has been found to be adulterated with roots of *Panax quinquefolius* L. (American ginseng) and *Eleutherococcus senticosusmaxim* (Siberian ginseng), which may cause health problem [164,165]. Another similar example is *Panax ginseng* being used as adulterant in roots of *Mandragora officinarum* L. (Solanaceae) because they are morphologically similar, but have completely different pharmacological effects and phytochemistry [166]. Further, roots of *Pfaffia panaculata* (Mart.) *Kuntze* (Amaranthaceae), also known as “Brazilian ginseng or suma root”, morphologically resembles *P. ginseng* roots, but the phytochemical content is different and, hence, pharmacological action is also different [167].
(e) Some other examples of physical similarity between species include the flower *Anthemisnobilis* L. and the chamomile, *Matricaria chamomilla* L. (Asteraceae), both of which are listed in the European Pharmacopoeia as therapeutic plants, as well as several other species in the family Asteraceae, such as *Tanacetum parthenium* (L.) Sch. Bip., *Tanacetum cinerariifolium* (Trevir.) *Schultz Bip*., *Tripleurospermum callosum* (Boiss. et Heldr.) *E. Hossain*, *Bellis perennis* L.and *Leucanthemum vulgare* L. Furthermore, the pharmacological activity differs depending on the phytoconstituents [168,169].
(f) One of the serious intentional adulterations is use of peanut skin extract in different grape products. Grape seed-containing drugs are said to have very high bioactive polyphenols content and used in prevention of cardiovascular and neurodegenerative disorders. The peanut skin is used as it is widely available, is a high-volume byproduct and is very cheap compared to grape products. However, it is a potential allergen, which may cause serious concerns when used as adulterant, which has been reported in a study where tested products contained no detectable quantities of grape seed extract, but only peanut skin as adulterant [170].
(g) One of the most important categories affected by adulteration is species containing essential oils (spices). As these spices are of high economic value and are export-oriented commodities, these are widely adulterated with natural and synthetic adulterants. One of the most commonly reported examples is adulteration of pure *Lavandula angustifolia* Mill. (Lamiaceae) oil with other species of same genus, which are almost six times cheaper. Essential oils from citrus are usually adulterated with sweet orange essential oil. Another quoted case is use of citronella oil (*Cymbopogon winterianus*), which is quite cheaper, as an adulterant in very high economic value essential oil from *Melissa officinalis* herb (balm oil). *Melissa officinalis* herb contains citronellal as the main constituent whereas citronella oil contains enantiomeric mixtures of citronellal [171,172].
(h) Another intention of adulteration is to add to the industrial value to the product. For example, synthetic α-irone and β-irone is added to iris (*Iris* sp., Iridaceae) oil to enhance the commercial value of the product, linalyl acetate or linalool, as the olfactory quality of bergamot or lavender oil becomes improved [171,172]. Other similar cases are when vegetable oils are added to increase the weight of other products—for example, lemongrass oil is diluted with coconut oil and sandalwood oil with polyethylene glycol. Leaf cinnamon essential oil contains lesser quantity of cinnamaldehyde but has the same olfactory notes as those of cinnamon bark essential oil, and, therefore, it has been reported as adulterant. Cheaper petit-grain oil made from leaves is used as adulterant in neroli oil made from the flowers of *Citrus aurantium* L. spp. *amara* L. var. *pumilia* (Rutaceae) [171]. Another case study reports that samples of the dietary supplements containing the leaf extract of *G. biloba*, used for cerebrovascular diseases, tinnitus and Alzheimer’s type of dementia, were demonstrated to be adulterated with free flavonols and glycones (such as quercetin and kaempferol, respectively) as well as genistein, the isoflavone derivative [173]. In another case, abietic acid has been reported as an adulterant in a herbal preparation used for the treatment of psoriasis [174].
(i) One potential hazardous adulteration that has harmful effects on human health is adulteration with allopathic drugs. The commonly used molecules in such types of adulterations are non-steroid anti-inflammatory drugs (NSAIDs), steroids, anti-diabetics and analgesics. For example, glibenclamide and metformin are reported in anti-diabeticherbal/botanical supplements [175,176,177]. In addition, it has been reported that herbal anti-diabetic remedies contain chlorpropamide, gliclazide, glimepiride, glipizide, pioglitazone, tolazamide and tolbutamide. Many analgesic compounds, including codeine, indomethacin, ketoprofen, morphine, oxyphenbutazone, paracetamol, phenylbutazone, diclofenac, dipyrone, ibuprofen, mefenamic acid, salicylamide and salicylic acid, are found in adulterated dietary supplements. It has been reported that herbal preparations contain anabolic steroids and hormones, such as rostendione, betamethasone valerate, betamethasone, clenbuterol, dexamethasone, flumethasone, hydrocortisone, prednisolone, prednisone, testosterone propionate, testosterone isocaproate, testosterone phenylpropionate and testosterone decanoate [178]. Another significant case that has been reported is adulteration with phosphodiesterase type-5 (PDE-5) inhibitor analogues, such as sildenafil citrate (Viagra^®^, Pfizer, New York, NY, USA), vardenafil hydrochloride (Levitra^®^, Bayer, Leverkusen, Germany), and tadalafil (Cialis^®^, Elli Lilly, Indianapolis, IN, USA), have been found in dietary supplements containing well-known natural constituents such as *Panax ginseng* L., *Astragalus membranaceus* (Fisch.) Bunge, *Schizandra chinensis* (Turcz.) Baill., *Ginkgo biloba* L., and many others. One of the possible consequences of such adulteration with prescription-only drugs such as phosphodiesterase type-5 (PDE-5) inhibitor analogues, which are contraindicated in men taking nitrates, may lead to unsafe drop in blood pressure. Such reported adulterations are homosildenafil in a food beverage; acetildenafil and hydroxyhomosildenafil in some other herbal products; vardenafil, sildenafil, tadalafil, and vardenafil in an herbal product sold in Hong Kong; benzamidenafil in herbal products; and thiosildenafil, a thioketone analogue of sildenafil, in herbal aphrodisiac supplements; nitroso-prodenafil, a prodrug of aildenafil, is also a reported adulterant, which is as carcinogenic as nitroso derivatives. Researchers examined 91 herbal items for the presence of PDE-5 inhibitors and found that 74 of them actually contained the PDE-5 inhibitor analogs, despite the fact that none of the labels mentioned the presence of a synthetic inhibitor. Eighteen of twenty-three herbal dietary supplements on the Dutch market tested positive for sildenafil or a comparable PDE-5 inhibitor in a similar investigation. [179,180].

Misleading labels are also one of the sources that lead to harmful effects on human health. In one study conducted on five samples of soy-based dietary supplements, the isoflavone content was analyzed and compared with claimed label checked. It has been reported that three of five supplements failed to contain the claimed amount of the isoflavones, i.e., genistein and daidzein. It has also been mentioned that one of the preparations that claimed to contain 60 mg of isoflavone based on genistein per tablet and was found to contain 1.538 mg of genistein *per* tablet after HPLC analysis [181].

## 5. Formulation Challenges

Drug interactions may be described as the situation when the activity of one active constituent is affected due to presence of other constituents. It may be food–drug interaction or drug–drug interaction. The pharmacological response may alleviate, lessen or induce side effects [182,183].

(a) Garlic (alicin) exhibits a hypotensive property and a hypocholesterolemic effect, acts as an anti-inflammatory agent and possess anti-bacterial as well as anti-fungal properties. When it is administered with anticoagulants (such as warfarin), it may lead to increased bleeding. With hypoglycemic drugs, such asinsulin or glipizide, it may cause hypoglycemia. With protease inhibitors (such as indinavir or saquinavir), garlic decreases their blood levels and effectiveness [184,185].
(b) Ginger is commonly used to treat various types of stomach problems—such as, to expel gas, formotion sickness, diarrhea, nausea (anti-emetic) and loss of appetite. It is also used in pain relief from arthritis, menstrual pain, upper respiratory tract infections—coughs and bronchitis. Ginger taken with anticoagulants may lead to risk of bleeding. If ingested with hypoglycemic drugs, such as insulin or glipizide, it may cause hypoglycemia. When co-administered with calcium channel blockers, ginger might reduce further or cause an irregular heartbeat [186,187].
(c) Green tea (polyphenols) improves mental alertness and thinking. It is also used to treat a plethora of other medical conditions, including Crohn’s disease, Parkinson’s disease, cardiovascular disease, diabetes, hypotension, chronic fatigue syndrome (CFS), tooth decay, kidney stones and skin conditions. Consuming green tea with stimulant medications could have dangerous consequences, such as elevated heart rate and blood pressure. Bortezomib (Velcade) may not be as effective against some cancers if used with green tea. Consuming green tea may reduce the effectiveness of warfarin [188,189].
(d) The leaf extract of *Gingko biloba* is effective in the treatment of Alzheimer’s disease and other forms of dementia, Raynaud’s syndrome, peripheral vascular disease, vertigo and dizziness, premenstrual syndrome (PMS) and improving color vision in people with diabetes. Ginkgo, when administered with anticoagulants/with NSAIDs, it may increase the risk of bleeding. When administered with anticonvulsants, it may reduce the effectiveness in preventing seizures [190,191].
(e) Licorice has been used for various digestive system complaints such as stomach ulcers, heartburn, colic and chronic gastritis. It is also used for sore throat, bronchitis and in treating infections caused by bacteria or viruses [192]. Licorice is also used in an herbal form called Shakuyaku-kanzo to increase fertility in women. It is also used to treat prostate cancer and the skin disorders such aseczema, in combination with other herbs. It may make antihypertensive drugs less effective, as it may increase salt and water retention. When taken with anti-arrhythmic drugs, it may decrease their efficacy as it may increase the risk of an abnormal heart rhythm. In some cases, it decreases the levels of potassium as it increases urine formation. In such cases, the risk of digoxin toxicity also increases, if patient is on digoxin [193,194,195].
(f) Kava root (kava-lactones) medicine, native to South Pacific, is used to calm anxiety, stress and to treat insomnia. It is also used in the treatment of attention deficit hyperactivity disorder (ADHD), depression, migraines and other headaches, chronic fatigue syndrome (CFS), epilepsy, psychosis, common cold and other respiratory tract infections, muscle pain, tuberculosis and cancer prevention. Kava is applied to the skin for some skin disorders such asleprosy, to promote wound healing. It is also used in urinary tract infections (UTIs), pain and swelling of the uterus, menstrual discomfort and hot flushes in women with menopause. It is also used as pain reliever in toothaches. When co-administered with barbiturates and benzodiazepines, it may prolong or intensify their effects [196,197].
(g) Chamomile (tea extract) is used as tea or dietary supplement for stomach cramps, to treat irritation from chest colds. It is also used for slow healing wounds, abscesses, gum inflammation, and skin conditions such as eczema, chickenpox and diaper-rash. The risk of bleeding increases when it is co-administered with anti-coagulants. Iron absorption also reduces in the presence of tea extract. The pollen is allergic in some cases. If one is allergic to ragweed pollen, chamomile use must be prohibited [198,199].

## 6. Patent Literature

Several published patents on nutraceutical applications in various areas, such as the agriculture and healthcare sector (such as promotion of health and disease prevention), and as nanocarriers for the improved delivery of nutraceuticalsas a support therapy for many diseases, have been summarized in Table 3. Many nutritional products are commercially available and used widely.

## 7. Conclusions and Future Prospects

To conclude, nutraceuticals area potentially growing sector and are engaged in both the fields, either medical treatment or nutrition so as to assure integrated medical assistance. These act as potential dietary supplements, prevention of diseases such as CVD, the support and treatment of various types of cancer, and other healthcare benefits. Therefore, nutraceutical industries now understand and perceive extensively about the potential success of nutrients that affect people in healthcare. At present, medical care is assessed to be the domain of drugs. On the contrary, nutrition is only appraised to be a product for healthy living. In the forthcoming years, it is anticipated that work will be performed, as they both interact and complement each other. The implementation of newer technologies such as the application of genetically modified technology in the food industry, nanotechnology-based nutraceuticals, etc., leads to better medical treatment and health care benefits, which further extended the increase in the nutraceuticals revenue market. The scientific research ratifies that the improved safety and potential effects of newly developed nutraceutical products will further stimulate the investments in newer technologies, such as nutrigenomics, converging techniques, varied imaging technologies and its applications in nutrition development and healthcare.

## Figures and Tables

**Table 1:** Clinical data findings on nutraceuticals.

Nutraceuticals	Bioactive Molecule	Dosage	Formulation	Duration of Study	Action	Population Size (Volunteers)	References
Omega-3	Eicosapentaenoic acid (EPA) and Docosahexaenoic acid (DHA)	60 mg/kg/day	Capsule	12 months	Cystic fibrosis	15	[13]
Omega-3	Eicosapentaenoic acid (EPA) and Docosahexaenoic acid (DHA)	900 mg in the omega-3 rich group and 250 mg in the omega-3 poor group	Capsule	15 days	Reduction of chemotherapy-related toxicities	61	[14]
Omega-3	Eicosapentaenoic acid (EPA) and Docosahexaenoic acid (DHA)	2000 mg/day	Capsule	21 days	Appetite	72	[15]
Aloe vera	aloe-emodin, aloin, aloesin, emodin, and acemannan	-	Gel	2 months	Acute dermatitis	120	[16]
Aloe vera	aloe-emodin, aloin, aloesin, emodin, and acemannan	60 mL	Juice	5 months	Irritable bowel syndrome	110	[17]
Seaweed	polysaccharides, proteins, lipids and polyphenols	2000 mg/d	Extract	12 weeks	High-density lipoprotein (hdl) cholesterol	34	[18]
Wheatgrass	vitamins, (A, B, C and E), minerals such as iron, calcium, magnesium, benzo(a)pyrene, ferulic, gallic, caffeic, syringic and p-coumaric acid	-	Cream	12 weeks	Plantar fasciitis	134	[19]
Wheatgrass	vitamins, (A, B, C and E), minerals such as iron, calcium, magnesium, benzo(a)pyrene, ferulic, gallic, caffeic, syringic and p-coumaric acid	100 cc/day	Juice	1 month	Active distal ulcerative colitis	23	[20]
Ginseng	ginsenosides	100 mg twice a day	Capsule	12 weeks	Psychomotor functions	16	[21]
Ginseng	ginsenosides	-	Hydrogel	2 weeks	Skin homeostasis	20	[22]
Echinacea	polysaccharides, glycoproteins, alkamides, cichoric acid, caftaric acid and chlorogenic acids	20 mg or 40 mg twice a day	Powder	6 weeks	Anti-anxiety and anti-depressant	104	[23]
Echinacea	polysaccharides, glycoproteins, alkamides, cichoric acid, caftaric acid and chlorogenic acids	5 mL	Oral Suspension	6 months	Tonsillitis	300	[24]

**Table 2:** List of nutraceuticals with health benefits.

Nutraceuticals/ Dietary Supplements	Nutrients	Health Benefits
Water Soluble Vitamins	Vitamin C	Wound healing, Antioxidant
	Vitamin B1	Carbohydrate metabolism, Neurological function
	Vitamin B2	Energy metabolism, Nerve function
	Vitamin B3	Brain function
	Vitamin B6	Convert proteins to energy
	Vitamin B12	Formation of RBC’s, Synthesis of amino acids Metabolism of fat, protein and carbohydrate
	Folic acid	Formation of RBC’s, Formation of genetic material of cells
	Pantothenic acid	Intraneuronal synthesis of acetylcholine Synthesis of cholesterol, steroids, and fatty acids
Fat Soluble Vitamins	Vitamin A	Cancer, Skin disorder, Healthy vision Antioxidant
	Vitamin D	Absorption of calcium, Formation of bones and teeth
	Vitamin E	Boost immune system, Antioxidant
	Vitamin K	Blood clotting
Minerals	Calcium	Maintaining bone strength, blood clotting
	Iron	Oxygen transport, Energy production
	Magnesium	Healthy nerve and muscle function and bone function
	Phosphorus	Phosphorylation process, Genetic material
	Copper	Heart functioning, Iron absorption
	Iodine	Functioning of thyroid gland
	Chromium	Diabetes
	Selenium	Antioxidant
	Zinc	Sperm production, wound healing
Herbals	Aloe vera	Anti-inflammatory, Wound healing
	Evening primrose oil	Treatment of atopic eczema
	Garlic	Anti-bacterial, Anti-fungal
	Ginger	Carminative, Anti-emetic
	Ginseng	Adaptogen
	Green tea	Cell mediated immunity, Antioxidant

**Table 3:** Patent Literature on nutraceuticals.

**Patent Number**	**Patent Title**	**Year**	**Reference**
CN104193540B	Fertilizer and insecticide use nutraceuticals disease prevention, disease prevention nutraceutical insecticidal fertilizer slow-release agent and the use thereof	2016	[200]
CN104262041A	Nutritional disease-preventing pesticide fertilizer and application thereof, nutritional disease-preventing pesticide fertilizer slow release agent and application thereof	2015	[201]
CN101371628A	Disease-preventing nutrient bag and manufacture method thereof	2009	[202]
US9669199B2	Bio-synchronous transdermal drug delivery for longevity, anti-ageing, fatigue management, obesity, weight loss, weight management, delivery of nutraceuticals and the treatment of hyperglycemia, Alzheimer’s disease, sleep disorders, Parkinson’s disease, aids, epilepsy, attention deficit disorder, nicotine addiction, cancer, headache and pain control, asthma, angina, hypertension, depression, cold, flu and the like	2017	[203]
WO2008054788A3	Transdermal delivery techniques for drugs, nutraceuticals and other active substances	2008	[204]
EP1835818B1	Product and method for producing a vehicle for oral administration of nutraceuticals	2011	[205]
US5955269A	Methods of screening foods for nutraceuticals	1999	[206]
EP2349302A4	Cardio-protective effects of nutraceuticals isolated from nigella sativa seeds	2012	[207]
US10517316B2	Combination of 25-hydroxyvitamin D and antioxidants/anti-inflammatories for bovine health	2016	[208]
US20080118583A1	Phyto-nutraceutical synergistic composition for Parkinson’s disease	2008	[209]
AU2005200614A1	Nutraceuticals for the treatment, protection and restoration of connective tissues	2005	[210]
US7494674B2	Nutraceutical with tart cherries and method of treatment therewith	2009	[211]
CN101534807A	Pharmaceutical and nutraceutical products comprising vitamin k2	2009	[212]
US20100063153A1	Anti-cholesterolemic compounds and methods of use	2010	[213]
US6630160B1	Process to modulate disease risk with doses of a nutraceuticals	2003	[214]
US6080788A	Composition for improvement of cellular nutrition and mitochondrial energetics	2000	[215]
US20160089411A1	Method and composition for treating symptoms of sickle cell disease	2016	[216]
CN102088995B	The use of angiogenin and angiogenin agonist for treating diseases and disorders	2016	[217]
US20080193590A1	Highly refined cellulose nutraceutical compositions and methods of use	2008	[218]
US20040009244A1	Composition comprising melissa leaf extract for anti-angiogenic and matrix metalloproteinase inhibitory activity	2004	[219]
US20110009360A1	Nutraceutical Composition and Methods for Preventing or Treating Multiple Sclerosis	2011	[220]
CN1822768A	Nutraceutical for the prevention and treatment of cancers and diseases affecting the liver	2006	[221]
US20210292265A1	Chalcones and derivatives for use in medicaments and nutraceuticals	2021	[222]
US20210113554A1	Nutraceuticals Having Sustained Release for Improved Bioavailability and Method of Production	2021	[223]
US20210290722A1	Nutraceuticals supplement composition for regulating metabolism and anti-aging	2021	[224]
US10981083B2	Process for fractionation and extraction of herbal plant material to isolate extractives for pharmaceuticals and nutraceuticals	2021	[225]
US20220040248A1	Prevention of Neuroinflammation associated Memory Loss Using Nutraceutical Compositions	2022	[226]
US11270791B2	In silico methods for obtaining nutraceutical compositions	2022	[227]
US20220160744A1	Multilayer pharmaceutical or nutraceutical solid dosage forms comprising pyrimidine and/or purine derivatives and b vitamins, preparation and uses thereof	2022	[228]
US20220184100A1	Enhanced d vitamin nutraceutical compositions and methods for making and used same	2022	[229]

## Data Availability

Not applicable.
